# Biochemical and Proteomic Characterization of Recombinant Human α/β Hydrolase Domain 6

**DOI:** 10.1038/s41598-018-36633-4

**Published:** 2019-01-29

**Authors:** Christina Miyabe Shields, Nikolai Zvonok, Alexander Zvonok, Alexandros Makriyannis

**Affiliations:** 1 Department of Pharmaceutical Sciences, Northeastern University, Boston, MA 02115 USA; 2 Center for Drug Discovery, Northeastern University, Boston, MA 02115 USA; 3 Department of Chemistry and Chemical Biology, Northeastern University, Boston, MA 02115 USA

## Abstract

Human alpha/beta hydrolase domain 6 (hABHD6) is an enzyme that hydrolyzes 2-arachidonoylglycerol (2-AG), a potent agonist at both cannabinoid CB1 and CB2 receptors. *In vivo* modulation of ABHD6 expression has been shown to have potential therapeutic applications, making the enzyme a promising drug target. However, the lack of structural information on hABHD6 limits the discovery and design of selective inhibitors. We have performed *E*. *coli* expression, purification and activity profiling screening of different hABHD6 constructs and identified a truncated variant without *N*-terminal transmembrane (TM) domain, hΔ29-3-ABHD6, as the most promising protein for further characterization. The elimination of the TM domain did not affect 2-AG or fluorogenic arachidonoyl, 7-hydroxy-6-methoxy-4-methylcoumarin ester (AHMMCE) substrates hydrolysis, suggesting that the TM is not essential for enzyme catalytic activity. The hΔ29-3-ABHD6 variant was purified in a single step using Immobilized Metal Affinity Chromatography (IMAC), in-solution trypsin digested, and proteomically characterized by Matrix Assisted Laser Desorption/Ionization Time-of-Flight Mass Spectrometry (MALDI-TOF MS). The *N*-terminal peptide without methionine was identified indicating on a post-translational modification of the recombinant protein. The mechanism of inhibition of hABHD6 with AM6701 and WWL70 covalent probes was elucidated based on MS analysis of trypsin digested hABHD6 following the Ligand Assisted Protein Structure (LAPS) approach. We identified the carbamylated peptides containing catalytic serine (Ser^148^) suggesting a selective carbamylation of the enzyme by AM6701 or WWL70 and confirming an essential role of this residue in catalysis. The ability to produce substantial quantities of functional, pure hABHD6 will aid in the downstream structural characterization, and development of potent, selective inhibitors.

## Introduction

The endocannabinoid system is a crucial regulator of mammalian homeostasis and since its discovery in the early 90’s, it has been investigated for its therapeutic applications^1,2^. The psychoactive component of the marijuana plant *Cannabis sativa*, Δ^9^-tetrahydrocannabinol, was the initial subject, but further research uncovered an entire endogenous system that is still expanding with new members. Currently the endocannabinoid system is comprised of two G-protein coupled receptors, cannabinoid receptor 1 and 2 (CB1 and CB2, respectively), two primary endogenous ligands, 2-arachidonoylglycerol (2-AG) and *N*-arichodonoylethanolamine (AEA), and the proteins involved in their synthesis, transport, and inactivation^1–3^.

Recently, in addition to known endocannabinoid-degrading enzyme monoacylglycerol lipase (MGL), a new enzyme, alpha/beta hydrolase domain 6 (ABHD6), has been found to break down between 4–40% of 2-AG, a potent agonist at both CB1 and CB2 receptors^4–6^. ABHD6 belongs to the alpha-beta hydrolase (ABHD) superfamily, a diverse class of serine hydrolases that is recognized by a common structural motif, the alpha-beta hydrolase fold, despite significant variability in primary amino acid sequence^7,8^. These enzymes all function through a highly conserved catalytic triad containing, serine, aspartic acid, and histidine residues (Ser^148^, Asp^278^, and His^306^ - respectively in the hABHD6).

While monoacylglycerol lipase (MGL) is a major player in deactivating endocannabinoid signaling by hydrolyzing presynaptic 2-AG not bound to cannabinoid CB1 receptor, ABHD6 has been shown to control separate pools of 2-AG and, thus, has a different therapeutic profile^5^. The biochemical, pharmacological and structural properties of MGL have been well characterized and selective and potent inhibitors have been reported. However, *in vivo* studies of promising MGL inhibitors have been shown to cause CB1 receptor desensitization due to the significant increase in concentration of 2-AG; this may lead to negative psychological side effects in human trial^9,10^. ABHD6 has been found to be located postsynaptically as an integral membrane protein with a single transmembrane (TM) domain^5^. In contrast to MGL, inactivation of ABHD6 by inhibition or disruption of ABHD6 protein expression causes a moderate increase in 2-AG levels without CB1-related side effects, making the enzyme a promising new drug target. Indeed, ABHD6 knockout studies have identified it as an important regulator of metabolism, modulating insulin secretion while preventing mice from developing obesity and diabetes^11–14^. Additionally, treatment with ABHD6-selective inhibitors has been shown to aid in the functional brain recovery of mice after traumatic head injury^15^. ABHD6 knock-out mice were viable and developed normally^11,12^, indicating that the complete inhibition of this enzyme at any stage of development does not cause serious detrimental side effects. This makes ABHD6 an attractive and unexplored endocannabinoid drug target with an expansive therapeutic profile that deserves further biochemical analysis.

## Materials and Reagents

Unless specifically mentioned, all standard laboratory chemicals were obtained commercially from Sigma Chemical Co. (St. Louis, MO) and Fisher Scientific (Pittsburgh, PA). The fluorogenic substrate AHMMCE was developed, synthesized, and purified at the Center for Drug Discovery, Northeastern University (Boston, MA) from commercial reagents by a synthetic route to be detailed elsewhere. Coomassie G-250 stain, Laemmli electrophoresis sample buffer, PVDF membrane, molecular weight markers and SDS-PAGE gels were from Bio-Rad (Hercules, CA). Trypsin Gold, MS grade, was purchased from Promega (Madison, WI). The Pierce 660 Protein Concentration Determination Kit was obtained from Thermo Fisher Scientific (Pittsburgh, PA).

## Methods

### pET45His6hABHD6, pET45His6hΔ29ABHD6 and pET26hΔ29ABHD6His6 Expression Constructs

The hABHD6 isoform 1 cDNA (OriGene Technologies Rockville, MD) was PCR amplified as full length or truncated variants and cloned into pET45b or pET26b plasmids for expression of protein with an *N*-terminal or *C*-terminal hexa-histidine tag.

#### pET45His6hABHD6 and pET45His6hΔ29ABHD6 constructs for expression of protein with an *N*-terminal hexa-histidine tag

The PCR products coding of a full-length hABHD6 or the first 29^th^ amino acids truncated hΔ29ABHD6 variant were obtained on the hABHD6 cDNA template with either forward 5′-ATAGGATCCATTGTGATTGCGGGCGGC or 5′-ATAGGATCCATGGCCTTCAGCACTGATAAG primer in pair with reverse 5′- ATTAGTCGACTCAGTCCAGCTTCTTGTTG primer (*Bam*HI and *Sal*I cloning sites are underlined) using a high-fidelity Advantage 2 DNA Polymerase (Clontech, Mountain View, CA). The PCR products and pET45b expression vector (Millipore, Billerica, MA) were digested with *Bam*HI and *Sal*I (Thermo Fisher Scientific); digested pET45b was subsequently treated with Antarctic phosphatase (New England Biolabs) to remove the 5′-phosphates from the linearized vector. The *Bam*HI and *Sal*I digested dephosphorylated pET45b vector and PCR fragments were purified by GeneJET PCR Purification kit (ThermoScientific), ligated at room temperature for 1 h using T4 DNA Ligase and transformed into NEBb10 *E*. *coli* cells.

#### pET26hΔ29ABHD6His6 construct for expression of protein with *C*-terminal hexa-histidine tag

The PCR conditions, preparation of pET26b plasmid (Millipore) and amplified DNA for cloning, ligation and transformation into *E*. *coli* cells were performed similar to described above. The PCR product formed using forward primer 5′-ATAGGATCCATGGCCTTCAGCACTGATAAG in pair with reverse primer 5′-TAATCTCGAGCTTCTTGTTGTTGTCTG (*Bam*HI and *Xho*I cloning sites are underlined) and coding the first 29^th^ amino acids truncated hΔ29ABHD6 variant were cloned into pET26b vector following digestion with *Bam*HI and *Xho*I restriction enzymes.

For each transformation, clones selected on Luria broth-ampicillin (pET45-constructs; Amp − 100 μg/mL) or Luria broth-kanamycin (pET26-construct; Km – 25 μg/mL) agar plates were used for plasmid DNA preparations (GeneJET Plasmid Miniprep Kit, Fermentas, Maryland). In-frame of pET45b or pET26b vector and ABHD6 DNA junctions as well as the coding sequence of the recombinant gene cassettes were confirmed by sequencing (SeqWright). The pET45His6hABHD6, pET45His6hΔ29ABHD6 and pET26hΔ29ABHD6His6 plasmid DNA were transformed into the *BL*21(DE3) *E*. *coli* strain (New England Biolabs) to express of full-length hABHD6 and first 29^th^ amino acids truncated hΔ29-ABHD6 variant.

### Construction of hΔ29-3-ABHD6 Variant

The pET45His6Δ29-3-hABHD6 construct was generated using the Stratagene QuickChange site-directed mutagenesis kit (Agilent Technologies, La Jolla, CA), the pET45His6hΔ29ABHD6 DNA, forward 5′-CAGCACTGATAAGAATCTACTGGCGGAGGACATTGG and reverse 5′-CCAATGTCCTCCGCCAGTAGATTCTTATCAGTGCTG mutagenesis primers. The sequencing confirmed pET45His6hΔ29-3ABHD6 plasmid DNA was transformed into the *BL*21(DE3) *E*. *coli* expression strain.

### Expression of the hABHD6 Variants

A single colony of *E*. *coli BL*21(DE3) cells transformed with either pET45His6hABHD6, pET45His6hΔ29-3ABHD6 or pET26hΔ29-ABHD6His6 DNA was inoculated into 10 mL of Luria broth-ampicillin or kanamycin medium (LB/Amp 100 μg/mL or LB/Kan 25 μg/mL) and grown overnight, shaking at 250 rpm, at 30 °C. The next morning, 5 mL of this culture was inoculated into 500 mL of LB/Amp or LB/Kan and incubation temperature was reduced to 18 °C. After allowing the culture to grow to an OD_600_ of 0.8, the expression was started by the addition of isopropyl-β-D-thiogalactopyranoside (0.33 mM) and was allowed to proceed for 8 hours. Cells were harvested by centrifugation at 8,000 × g for 10 min at 4 °C, washed once with PBS, and stored at −80 °C for further analysis.

### *E*. *coli* Membrane Fraction Preparation and hABHD6 Solubilization

A cell pellet with full-length hABHD6 (2.5 g) was resuspended in 50 mL of lysis buffer containing 150 mM NaCl, 50 mM Tris, pH 8.0 (Lys1). The cells were lysed on ice by pulse sonication using three, 50 s sonication cycles: 1 s sonication bursts at 50 W power separated by 5 s intervals (Vibra-Cell 500 W, Sonics, Newtown, CT). The lysate was centrifuged at 4,000 × g for 20 min at 4 °C to pellet cell debris. The resulting supernatant was centrifuged at 145,000 × g for 45 min at 4 °C and the pellet was resuspended in Lys1 buffer. This was repeated twice for a total of three high-speed centrifugations collecting the final membrane fraction. The membrane fraction was resuspended in 5 mL of Lys1 buffer containing 1% v/v Triton X-100 and allowed to solubilize at 4 °C for 1 hr. The solution was centrifuged at 20,000 × g for 10 min at 4 °C and the supernatant containing hABHD6 was collected.

### hΔ29-3-ABHD6 Immobilized Metal Affinity Chromatography (IMAC) Purification

A cell pellet with hΔ29-3-ABHD6 (2 g) was resuspended in 25 mL of Lys1 buffer containing 1% Triton X-100 and sonicated on ice using three, 50 second sonication cycles detailed above. The lysate was rotated at 4 °C for 1 hr to complete extraction and solubilization of proteins from cells debris and then centrifuged at 20,000 × g for 10 min at 4 °C. The supernatant was diluted 1:1 with Lys1 buffer containing 20 mM imidazole and incubated with 200 μL (bead volume) of pre-equilibrated BD Talon metal-affinity resin (Clontech, Mountain View CA) for 1 hr at room temperature. The suspension was transferred to a gravity-flow column and allowed to settle. The resin was washed twice with 20 mL of Lys1 buffer containing 0.5% Triton X-100, and 10 mM imidazole, and once with Lys1 buffer containing 0.5% Triton X-100 and 25 mM imidazole. Purified hΔ29-3-ABHD6 was eluted with six 200 μL elutions of Lys1 buffer containing 0.5% Triton X-100, 10% v/v glycerol, and 250 mM imidazole.

### SDS-PAGE and Western Blotting

Protein samples and ladders (Bio-Rad) were denatured at room temperature for 20 min in Laemmli buffer containing 5% β-mercaptoethanol and resolved on AnyKD SDS-PAGE gels (Bio-Rad). Gels were either stained using Commassie blue (Bio-Rad) or proteins transferred to polyvinylidene fluoride membranes (PVDF) for immunodetection according to the QIAexpress Detection and Assay Handbook using a 1:1,000 dilution of anti-mABHD6 (generously provided by Dr. Brown Laboratory, Cleveland, OH) and a 1:10,000 dilution of anti-rabbit secondary antibody. Proteins were visualized using the ECL Western Blotting Analysis System (GE Healthcare, Piscataway, NJ). A FluorChem Imaging System (Alpha Tech Corp., San Leandro, CA) was used to photograph developed gels and blots.

### The hABHD6 Enzyme Activity Assays

The enzymatic parameters of substrate hydrolysis by generic hABHD6 enzyme were evaluated in three different biochemical assays: hydrolysis of 2-AG to AA and glycerol was quantified by either HPLC (1) or a glycerol-based colorimetric method (2), and a fluorometric method using the fluorogenic substrate AHMMCE (3). Prior enzyme assays a total protein concentration in supernatant of solubilized membrane fraction containing of full-length hABHD6 was determined using the Pierce 660 Kit (Thermo Fisher Scientific, Pittsburgh, PA). The assays to measure protein concentrations, intensity of fluorescence or color of formed products were performed in a 96-well plate format using a Synergy HT (BioTek Instruments, Winooski, VT) or EnVision (Perkin Elmer, Waltham, MA) plate readers. All activity assays were performed in triplicate and the Michaelis-Menten kinetic parameters were calculated using Prism software, Version 5 (Graphpad, San Diego, CA).

#### An HPLC assay with 2-AG substrate

The first method quantifies of the AA product formation by HPLC as previously detailed^16^. Briefly, 2-AG at various concentrations from 12.7 to 250 μM was incubated with 1 μg of supernatant of solubilized membrane fraction containing full-length hABHD6 in 150 mM NaCl, 50 mM Tris, pH 7.6 and 1% w/v BSA buffer for 1 hr at 37 °C. Samples were diluted 1:4 with acetonitrile and centrifuged at 20,000 × g for 5 min. A 20 μL aliquot of the supernatants were injected on an XDB-C18 HPLC column (4.6 × 50 mm, 3.5 μm) and substrate/product were separated under chromatographic conditions previously detailed^17^. A standard curve of both 2-AG and AA were run to quantify the amount of AA produced with external standards.

#### A colorimetric assay with 2-AG substrate

A previously described ABHD6 glycerol-based colorimetric assay^6^ based on a Cayman kit (Cayman Chemicals, Ann Arbor, MI) was employed to determine a quantity of glycerol formed during enzymatic 2-AG hydrolysis. Reaction mixtures in 50 mM sodium phosphate, pH 7.2 buffer (100 μL) containing 2-AG at various concentrations from 12.7 to 350 μM and supernatant of solubilized membrane fraction with full-length hABHD6 (1 μg of a total protein) were allowed to proceed for 30 min at room temperature followed by the addition to the wells of 100 μL of the Glycerol Enzyme Mixture containing 0.4 U/mL glycerol kinase, glycerol phosphate oxidase, and horse radish peroxidase. The plate was incubated for another 30 min at room temperature before the absorbance at 530 nm was measured and the glycerol produced quantified.

#### A fluorometric assay with AHMMCE substrate

A fluorometric assay based on the substrate AHMMCE, previously described for hMGL^16–19^ was optimized for hABHD6. Briefly, a supernatant of solubilized membrane fraction containing of full-length hABHD6 (1 μg of total protein in each well of a 96-well Costar 3650 plate) in 50 mM Tris-HCl, pH 7.6 assay buffer (AB) was incubated at specified concentrations (0.97, 1.85, 3.57, 6.89, 13.31, 25.72, 49.65, 95.86 μM) of AHMMCE for 1 hr at room temperature before being read every 15 min for another 3 hr incubation following excitation at 360 nm and emission at 460 nm (λ_ex_360 nm/λ_em_460 nm). The relative fluorescence units were converted to the concentration of 7-hydroxy-6-methoxy-4-methylcoumarin (HMMC) formed based on a standard curve of HMMC. Data was normalized relatively to a negligible non enzymatic AHMMCE hydrolysis and used for calculation of the hABHD6 enzyme kinetic parameters.

### Fluorometric hABHD6 Inhibition Assays Using AHMMCE Substrate

All inhibition assays were performed in a 96-well plate (Costar 3650) format in triplicate and the inhibition constants were calculated using Prism software, Version 5 (Graphpad, San Diego, CA).

#### Three point concentration assays

To determine compounds’ potencies and ranges of ABHD6 inhibition we conducted three point concentration assays. A solubilized membrane fraction containing of full-length hABHD6 (1 μg of total protein in each well) in AB was mixed with the compound dissolved in 50% DMSO/50% AB (bringing the volume to 196 μL and the final concentrations of the compound to 1, 10 and 100 μM). These samples were incubated for 15 min at room temperature and an AHMMCE stock solution (4 μL of 1 mM in DMSO, final concentration of substrate 20 μM) was added. The reaction was allowed to proceed at room temperature for 1 hr before being read at λ_ex_ 360 nm/λ_em_ 460 nm every 15 min for another 3 hr incubation. A standard curve of known concentrations of HMMC was used to convert the relative fluorescence of product into quantifiable concentrations to determine a range of ABHD6 inhibition.

#### Eight point concentration assays

For all compounds that inhibited >50% ABHD6 activity at concentration of 1 μM, full inhibition curves using eight different concentrations of inhibitor (8 point assay) were generated. To determine *IC*_50_ value of promising inhibitors the hABDH6 fluorometric inhibition assay, similar to MGL assay^17–19^ was developed. The 96 wells plate containing a solubilized membrane fraction of full-length hABHD6 (1 μg of total protein in each well) and a specified concentrations of the compound in AB (196 μL a total volume) was incubated for 15 min at RT. The fluorogenic substrate AHMMCE was added in each well (4 μL 1 mM, final concentration of substrate 20 μM) and the plate was incubated at RT for 1 hr before reading were taken every 15 min for another 3 hr incubation following λ_ex_ 360 nm/λ_em_ 460 nm. Data was normalized relatively to negligible non enzymatic AHMMCE hydrolysis. A standard curve of known concentrations of HMMC was used to convert the relative fluorescence of products into quantifiable concentrations used for *IC*_50_ calculation.

### MALDI-TOF MS Analysis of the hABHD6 Trypsin Digested Samples

IMAC purified samples of the hΔ29-3-ABHD6 protein (~5 μg, 30 μL) were desalted using a Bio-Spin 6 column (Bio-Rad) with 25 mM ammonium bicarbonate buffer, pH 8.0, containing 0.05% octyl-β-glucoside. The desalted samples were reduced and alkylated at room temperature in the dark with DTT (1 μL of 500 mM) for 15 minutes and IAM (5 μL of 550 mM) for 1 hr, respectively, and overnight digested with MS-grade trypsin (200 ng, “Trypsin Gold,” Promega) at 37 °C. Digested samples were co-crystallized with α-cyano-4-hydroxycinnamic acid matrix by spotting 0.5 μL of sample and 0.5 μL of 5 mg/mL matrix prepared in acetonitrile: water (1:1) with 0.1% trifluoroacetic acid onto an Opti-TOF 384-well plate insert. Spectra of tryptic hABHD6 peptides were acquired on a 4800 MALDI TOF/TOF mass spectrometer (Applied Biosystems, Foster City, CA) fitted with a 200-Hz solid state UV laser (wavelength 355 nm) in reflectron mode. All MS spectra of the tryptic digests were externally calibrated using a mixture of peptide standards (des-Arg1-bradykinin at MH^+^ 904.4681, angiotensin I at MH^+^ 1296.6853, Glu-fibrino peptide at MH^+^ 1570.6774, ACTH (clip 1–17) at MH^+^ 2093.0867, ACTH (clip 18–39) at MH^+^ 2465.1989 and ACTH (clip 7–38) at MH^+^ 3657.9294). The maximum allowable error was 50 ppm. Theoretical molecular weights of expected peptides after *in silico* trypsin digestion were calculated using FindPept and FindMod tools (ExPASy Server, Swiss Institute of Bioinformatics, Geneva, Switzerland).

### Inactivation of the hABHD6 by Inhibitors and Sample Preparation for MALDI-TOF MS Analysis

Purified hΔ29-3-ABHD6 (~5 μg, 30 μL, 4.5 μM) in buffer (150 mM NaCl, 50 mM Tris-HCl, 250 mM imidazole, 10% v/v glycerol, pH 8.0) was incubated for 1 hr at room temperature with or without covalent inhibitor (AM6701 or WWL70) at a 1:10 enzyme:inhibitor molar ratio. After evaluation of hABHD6 inactivation by AM6701 or WWL70 in fluorescent assay, the protein samples were reduced and alkylated at room temperature in the dark with DTT (1 μL of 500 mM) for 15 minutes and IAM (5 μL of 550 mM) for 1 hr, respectively. The samples were desalted with a Bio-Spin 6 Column in 50 mM ammonium bicarbonate buffer containing 0.05% w/v octyl-β-glucoside, pH 8.0. The desalted sample was digested with trypsin (200 ng) overnight at 37 °C.

## Results and Discussion

The structural characterization of the hABHD6 protein is critical for the successful development of novel, selective and potent enzyme inhibitors. The first step to achieve this goal is the establishment of a reliable expression system and purification method for the generation of active, pure and stable hABHD6 protein. Expression in *E*. *coli* is optimal in order to maximize the total yield of protein, however obtaining of active hABHD6 with enzymatic parameters similar to the wild type is essential and may compromise in prokaryotic host. The fact that neither glycosylation^20^ nor any other post translational modifications were observed and required for hABHD6 enzyme activity was considered as a distinct advantage for choosing the *E*. *coli* expression system. Several plasmid constructs expressing the hABHD6 variants in *BL*21(DE3) *E*. *coli* cells were evaluated to establish conditions of obtaining a pure functional protein in sufficient amounts for biochemical/pharmacological/proteomic characterization, assay development, and inhibitor screening.

### Engineering, Expression, and Single-Step IMAC Purification of the hABHD6 Variants

PCR amplified full length or truncated hABHD6 DNA was cloned into pET45b or pET26b plasmids for expression of the hABHD6 proteins with an *N*-terminal or *C*-terminal hexa-histidine tag, respectively.

The presence of an *N*-terminal hexa-histidine tag preceding an enterokinese cleavage site either in a full length or in the first 29^th^ amino acids truncated hABHD6 variant expressed in pET45-based constructs provided an opportunity for the downstream protein purification by IMAC and the option for tag removal.

**Figure 1:**
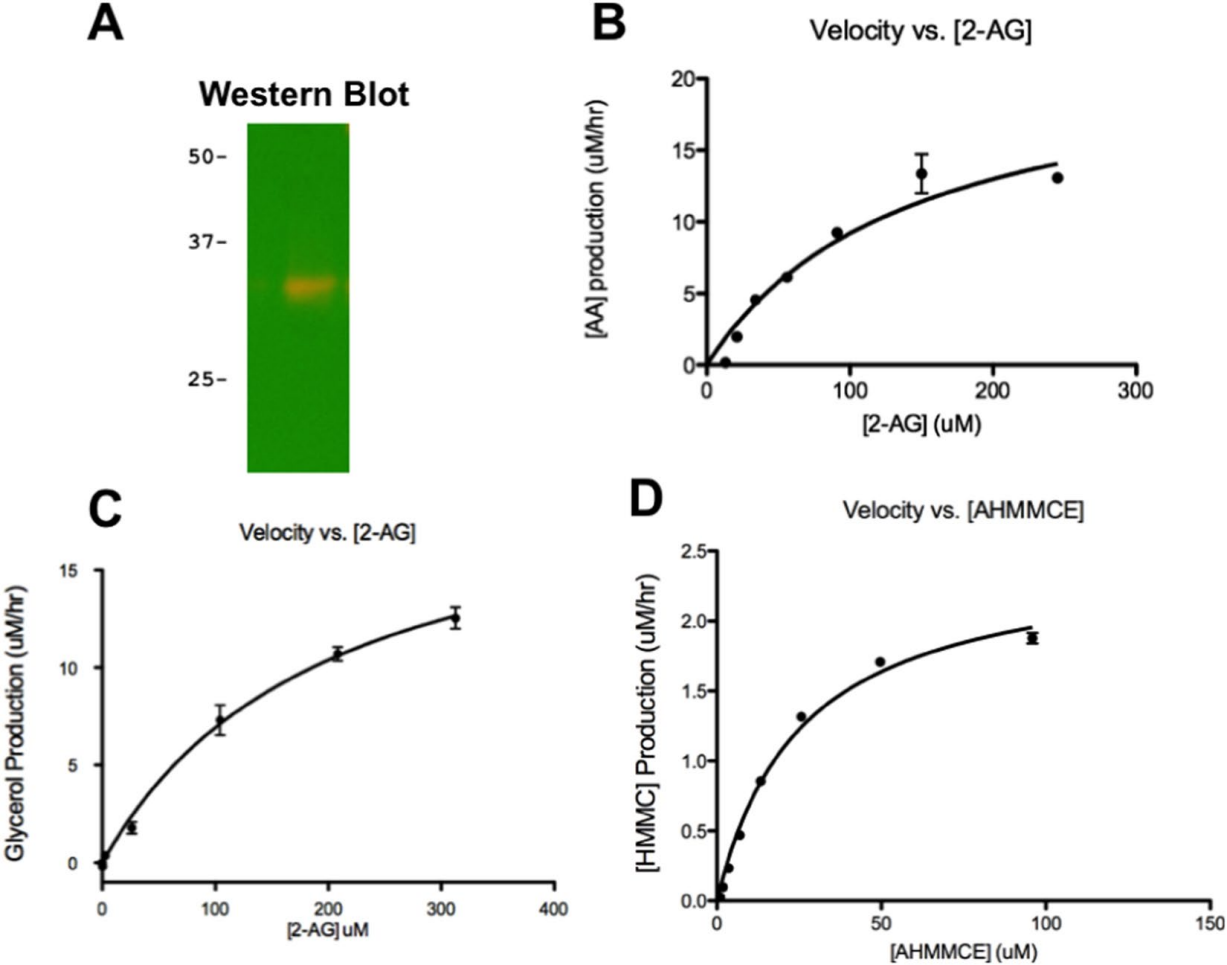
Full-length recombinant hABHD6 enzyme characterization. (**A**) Western Blot identification of recombinant hABHD6 using anti-mABHD6 antibody. Enzymatic parameters of hABHD6 determined with 2-AG substrate in: (**B**) HPLC activity assay and (**C**) Colorimetric glycerol-production activity assay. (**D**) Fluorescent activity assay with AHMMCE substrate.

The hABHD6 expression in *BL*21(DE3) *E*. *coli* cells was verified by Western Blot using anti-mABHD6 antibodies (Fig. 1A; mABHD6 and hABHD6 share a 94% sequence identity). The hABHD6 protein migrated faster than its predicted ~39 kDa molecular mass in SDS PAGE. This is most likely due to the presence of a TM domain, as observed with other integral membrane proteins^21^. The hABHD6 with the native TM domain was expressed predominantly as inactive protein accumulated in inclusion bodies (~95%, unedited Fig. S1 in supporting information). The active enzyme (~5%) was associated with the membrane fraction and was IMAC purified either directly from *E*. *coli* cells lysate or after membrane preparation and solubilization (Supplemental Fig. S1). However, the quality and quantity of purified hABHD6 protein was insufficient for comprehensive mass spectrometric studies.

**Figure 2:**
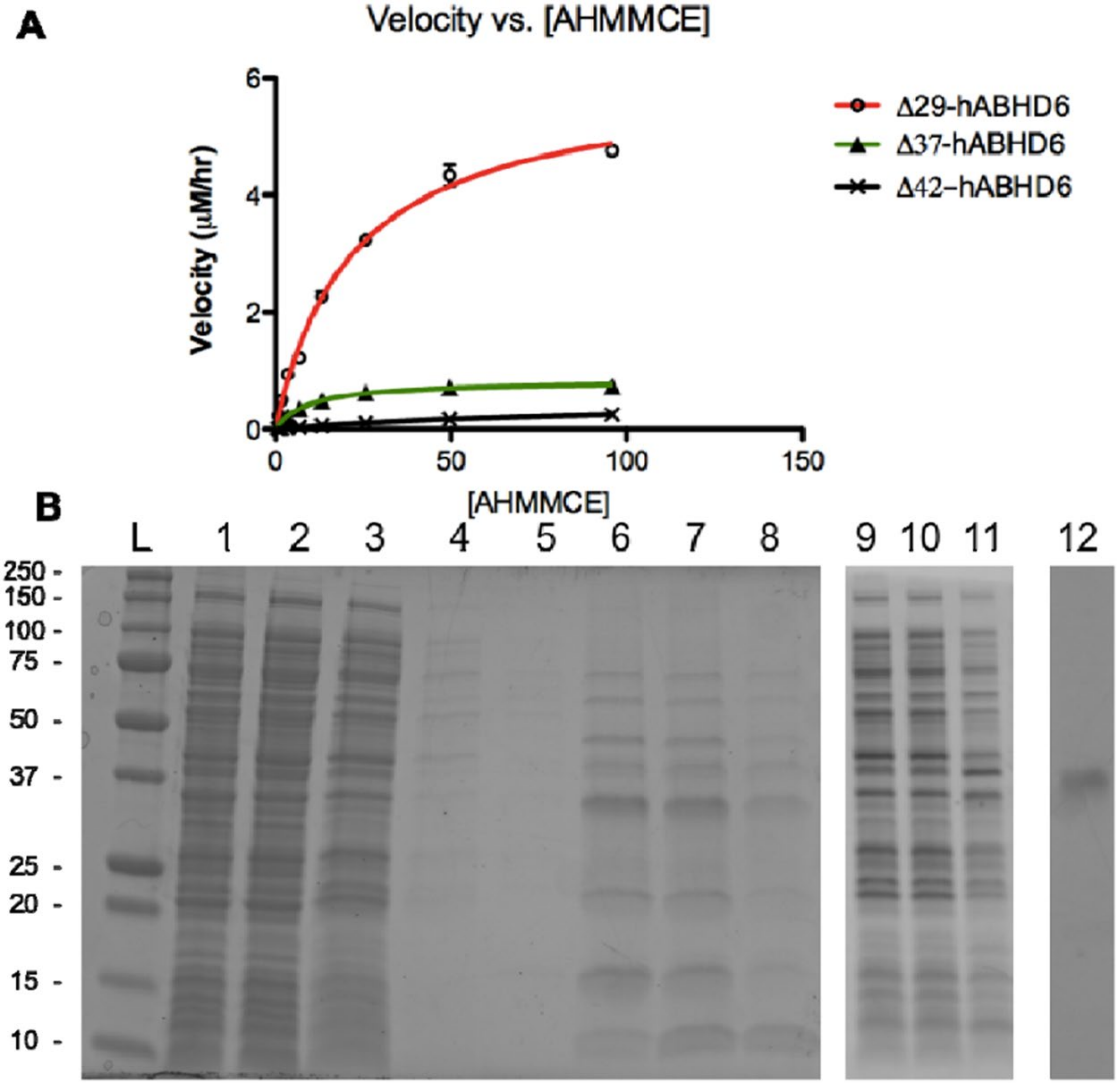
Recombinant Δ29, 37 and 42 hABHD6 truncated variants characterization. (**A**) Activity of the hABHD6 TM truncated variants in the fluoroscent assay. (**B)** SDS-PAGE analysis of purification of hΔ29-ABHD6: using Talon Co^2+^ resin, (1) - soluble cells lysate, (2) - unbound to resin fraction, (3–5) – resin washes 1–3, (6–8) - imidazole elutions 1, 3 and 5; and using cOmplete Ni^2+^ resin, (9–11) - imidazole elutions 1, 3 and 5, (12) - Talon resin 200 mM EDTA wash following imidazole elutions.

To improve the expression and purification of soluble hABHD6, the *N*-terminal TM domain of the protein, considered as not essential for the enzyme catalytic activity but complicated both soluble expression and purification, was chosen for deletion. Three *N*-terminal truncated variants without the first 29, 37, and 42 amino acid residues in hABHD6 were chosen for expression (Fig. 2A). All *N*-truncated hABHD6 variants were expressed at comparable level and exhibited a lower association with membrane as compared to the wild type hABHD6 protein. Truncation of the first 29^th^ amino acid residue (hereafter referred to as hΔ29-ABHD6) generated an enzyme with the highest activity (Fig. 2A; the detailed characterization of truncated mutants will be presented elsewhere).

The hΔ29-ABHD6 purification in a single-step IMAC procedure provided protein of approximately 30–40% purity in a mixture with other proteins (Fig. 2B). The X-Tractor buffer (Clontech, Mountain View, CA) and octyl-β-glucoside (OBG) detergent were both evaluated for purification of the hΔ29-ABHD6 following extraction from the cells, but the same co-purified proteins were presented in the eluted fractions (Supplementary Fig. S3). Our efforts to remove the co-purified proteins by increasing the imidazole concentration in the wash buffer were unsuccessful. We determined that even at low, such as 50 mM imidazole concentration in wash buffer, the hΔ29-ABHD6 protein was partially eluted from IMAC resin, suggesting a weak interaction of the *N*-terminal hexa-histidine tag and the tetradentate chelated Co^2+^ on Talon resin (Supplemental Fig. S2A). A significant ABHD6 activity was retained in the unbound fraction (Supplemental Fig. S2B), indicating that the *N*-terminal hexa-histidine tag may have been occluded from proper interacting and binding to the resin. To address this difficulty, we generated a construct expressing the hΔ29-ABHD6 protein with a *C*-terminal hexa-histidine tag (hΔ29-ABHD6His6). However, the quality of IMAC purified ABHD6 proteins with either *C*-terminal or *N*-terminal hexa-histidine tag were very similar (Supplemental Fig. S2C). Interestingly, a significant amount of active hΔ29-ABHD6 protein with either *N*- or *C*-terminal hexa-histidine tag was retained bound to the IMAC resin after additional multiple washes with 250 mM imidazole and was eluted only after stripping of the metal ions from the resin with 200 mM ethylenediaminetetraacetic acid (EDTA) (Figs 2B and S3).

**Figure 3:**
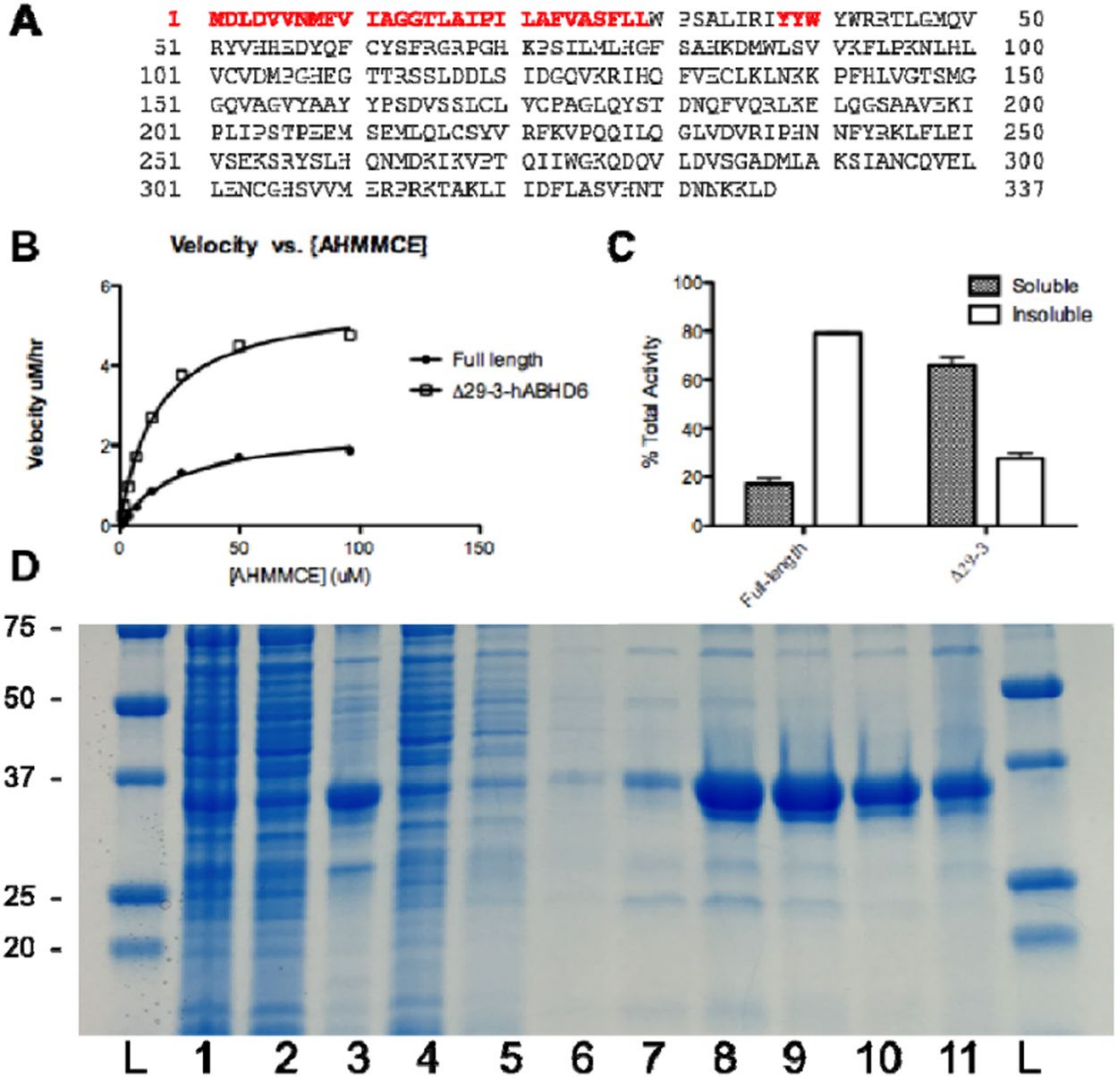
The hΔ29–3-ABHD6 variant characterization. (**A**) Primary sequence of full-length hABHD6 and the hΔ29-3-ABHD6 variant (deleted amino acid residues are marked in red). (**B**) Comparison of activity of full-length and hΔ29-3-ABHD6 variant using fluorogenic substrate AHMMCE. (**C**) Sub-cellular localization of full-length and hΔ29-3-ABHD6 variants based on activity profile. (**D**) Purification of hΔ29-3-ABHD6. Lanes are: (1) - total lysate, (2) - soluble fraction, (3) - insoluble fraction, (4) - unbound to resin fraction, (5–7) - resin washes 1–3 and (8–11) - elutions 1–4.

In a further attempt to improve the purification of hABHD6, the partial deletion of a unique aromatic cluster, Y^38^YWY^41^W, particularly the tyrosine residues which may be interfering with binding of the hexa-histidine tag to IMAC resin, was employed. Because tryptophan has been shown to be an important residue in serine hydrolases, including hMGL^22^, a deletion of Y^38^YW while preserving a single tyrosine and tryptophan residue (Y^41^W), was performed to create the final variant hereafter referred to as hΔ29-3-ABHD6 (Fig. 3A). The hΔ29-3-ABHD6 protein was expressed in *BL*21(DE3) *E*. *coli* strain and after cells lysis the majority of enzyme activity was mostly associated with the soluble fraction as opposed to the membrane fraction (Fig. 3C).

The hΔ29-3-ABHD6 enzyme performed comparably to the full-length native hABHD6 and hΔ29-ABHD6 variant with a *K*_m_ for AHMMCE of 20 μM (Fig. 3B), indicating that the binding pocket of the enzyme was not affected by either truncation or mutation. Moreover the maximal velocity of the hΔ29-3-ABHD6 variant to hydrolyze AHMMCE was twice higher compare to the enzyme with native TM domain intact. The remaining activity seen in the membrane fraction of hΔ29-3-ABHD6 is likely due to membrane-proximal behavior of the lid domain, which was not altered during truncation. Most importantly, hΔ29-3-ABHD6 was able to be purified by single step of IMAC with greater than ~90% purity (Fig. 3D).

### The hABHD6 Enzyme Activity Assays

We used two different biochemical activity assays with 2-AG substrate to verify that recombinant hABHD6 protein expressed in a prokaryotic system had the catalytic properties similar to the native mammalian enzyme. The first, an HPLC-based assay quantifying the formation of AA product from ABHD6 enzymatic hydrolysis of 2-AG showed a *K*_m_ of 144 μM and a *V*_max_ of 24 μM/hr (Table 1, Fig. 1B). In another, a previously published enzyme-linked colorimetric assay quantifying the formation of glycerol from 2-AG enzymatic hydrolysis, showed a *K*_m_ of 200 μM and a *V*_max_ of 21 μM/hr (Table 1, Fig. 1C). Obtained *K*_m_ and *V*_max_ values are comparable to previously published data utilizing ABHD6 overexpressed in mammalian cells and assayed with the native 2-AG substrate^6^. This confirmed that recombinant hABHD6 produced in prokaryotic expression systems behaves similar to native enzyme and can be used in our experimental studies.

**Table 1:** Enzyme Kinetics of Full Length ABHD6 and Δ29-3-hABHD6 Variant.

Enzyme Assay (Substrate)	Full-length hABHD6 ± SEM		hΔ29-3-ABHD6 ± SEM	
	*K*_m_ (μM)	*V*_max_ (μM/hr/mg)	*K*_m_ (μM)	*V*_max_ (μM/hr/mg)
HPLC (2-AG)	144 ± 10	24 ± 0.49	139 ± 4.0	21 ± 0.24
Colorimetric Gycerol (2-AG)	200 ± 38	21 ± 1.92	N/A	N/A
Fluorescent (AHMMCE)	26 ± 1.2	2.5 ± 0.07	20 ± 1.7	5.7 ± 0.04

All enzyme assays were performed three times in triplicate.

### Fluorometric hABHD6 Inhibition Assays Using AHMMCE Substrate

Both HPLC and colorimetric assays are suitable for characterization and evaluation of the recombinant ABHD6 enzyme activity. However, neither of these assays with the native 2-AG substrate is ideal for the rapid screening of compound libraries for the discovery of novel ABHD6 inhibitors. The HPLC-based assay is tedious, complicated, and requires prolonged instrumentation and data processing time. Although the glycerol-based assay is performed on a 96-well plate and is run much faster, the presence in samples of three other enzymes necessary for detection and quantification of glycerol creates the possibility of false positive identification of compounds as ABHD6 inhibitors and, therefore, may complicates screening process. To address these issues, especially in increasing the high-throughput capability, an activity assay based on the fluorogenic substrate AHMMCE was developed. The fluorescent assay with AHMMCE, previously introduced and used successfully for recombinant hMGL^16,17^ characterization and inhibitors discovery, was slightly modified for ABHD6 enzyme. The *K*_m_ and *V*_max_ parameters were determined to be 26 μM and 2.5 μM/hr, respectively (Table 1, Fig. 1D). This fluorescent assay provides a fast, convenient and efficient procedure for the evaluation of hABHD6 activities with or without inhibitors.

Screening of a large number of compounds to determine their potencies and ranges of ABHD6 enzyme inhibition was conducted in three points assay with inhibitors at concentration 1, 10 and 100 μM. For compounds that inhibited at least half of hABHD6 activities at concentration of 1 μM, full inhibition curves and *IC*_50_ values were generated using eight specified concentrations of inhibitor (8 point assay). From our compound library two inhibitors, AM6701 and WWL70 were selected for Ligand Assisted Protein Structure (LAPS) studies of the hABHD6. Apparent *IC*_50_ values of ABHD6 inhibition by AM6701 (0.9 nM) and WWL70 (285 nM) were determined in our fluorescent 8 point assay (Supplementary Fig. S4A,B). Interestingly, the *IC*_50_ value of ABHD6 inhibition by WWL70 using assay with fluorogenic AHMMCE substrate was 4 fold higher compare to reported *IC*_50_ value obtained in Activity-Based Proteome Profiling (ABPP) assay (70 nM) with the fluorescent rhodamine fluorophosphonate probe (FP-rhodamine)^23^. To address this WWL70 *IC*_50_ discrepancy between these two assays, we investigated ABHD6 inhibition by FP-rhodamine probe using a fluorescent assay with AHMMCE substrate. The apparent *IC*_50_ value of hABHD6 inhibition by FP-rhodamine was 10–20 fold higher compare to hMGL and hFAAH *IC*_50_ values determined in the similar fluorescent assays (3, 0.28 and 0.16 μM, respectively; data not presented). Therefore the *IC*_50_ values determined by ABPP profiling may provide lower *IC*_50_ values for hABHD6 inhibition due to insufficient concentration of probe (working FP-rhodamine concentration is 2 μM) to compete with inhibitor. As an example, a low affinity FP-rhodamine probe competed with WWL70 for ABHD6 enzyme active site in ABPP assay less effectively, providing to this inhibitor more competitive and potent *IC*_50_ value than in the fluorescent assay with AHMMCE substrate (70 nM and 285 nM, respectively). In addition, the ABPP assays were performed with mouse brain membrane preparations, a highly complicated mixture containing both lipids and other membrane proteins that could affect ABHD6 inhibition by FP-rhodamine.

### MALDI-TOF MS Peptide Fingerprinting of Trypsin Digested Native or Inhibitor Treated hABHD6

**Figure 4:**
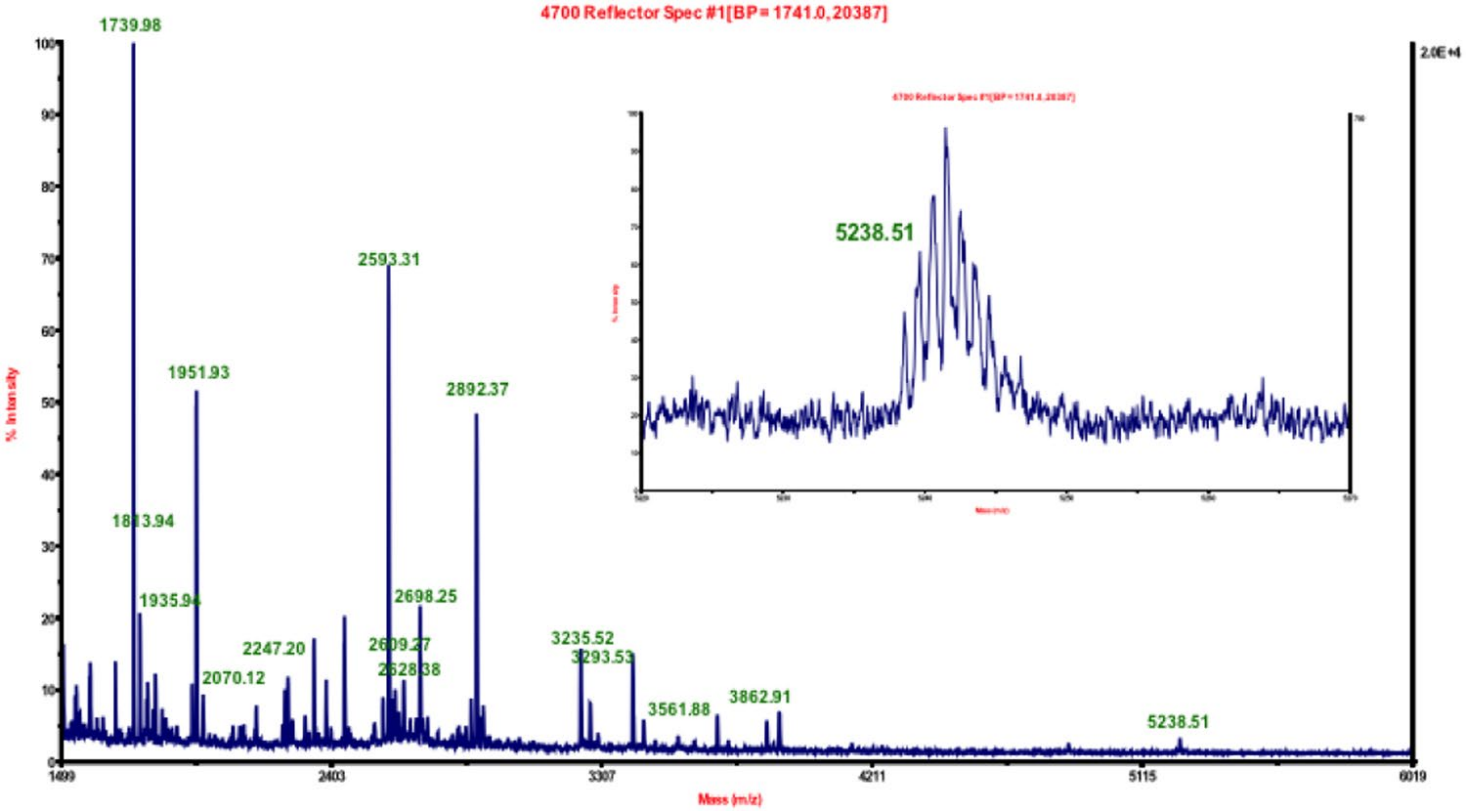
MALDI-TOF MS analysis of hΔ29–3-ABHD6 digested with trypsin. Specific peptides that matched with masses of *in silico* digesed hΔ29-3-ABHD6 protein are labeled in green. High-resolution MS spectrum of the precursor ion (*m/z* 5238.51) with the active site serine residue Ser^148^ is shown in inset.

Purified hΔ29-3-ABHD6 protein was subjected to in-solution trypsin digestion and the resulting peptides were fingerprinted using a 4800 MALDI TOF/TOF mass spectrometer (Fig. 4). The observed experimental masses of peptides were matched with the theoretical masses of peptides created using an *in silico* trypsin digestion of the hΔ29-3-ABHD6 protein. Tryptic peptides of the hΔ29-3-ABHD6 digest in mass range of 970–5239 Da and accuracy below 45 ppm error are presented in Table 2. Only small peptides (4 or less amino acid residues) were missed, while all other peptides covering 95% of the entire sequence of the hΔ29-3-ABHD6 protein were identified (Table 2).

**Table 2:** Specific Peptides Identified by MALDI-TOF MS.

Observed mass	Expected Mass	Error (ppm)	Peptide Sequence	Position*	Modifications	Missed Cleavages
3235.52	3235.48	−12.3	AHHHHHHVGTGSNDDDDKSPDPWPSALIR/(I)	1–29		1
2892.37	2892.346	−8.4	(R)/RTLGMQVRYVHHEDYQFCYSFR/(G)	34–55	CAM	2
2070.12	2070.118	−1	(R)/GRPGHKPSILMLHGFSAHK/(D)	56–74		0
3561.88	3561.897	4.8	(R)/GRPGHKPSILMLHGFSAHKDMWLSVVKFLPK/(N)	56–86	2 MSO TPO	2
977.52	977.512	−7.7	(K)/DMWLSVVK/(F)	75–82		0
1935.94	1935.916	−12.2	(K)/NLHLVCVDMPGHEGTTR/(S)	87–103	CAM	0
1951.93	1951.911	−9.6	(K)/NLHLVCVDMPGHEGTTR/(S)	87–103	CAM MSO	0
3293.53	3293.589	17.8	(K)/NLHLVCVDMPGHEGTTRSSLDDLSIDGQV K/(R)	87–116	CAM	1
1329.77	1329.71	−45.0	(K)/RIHQFVECLK/(L)	117–126	CAM	1
5238.51	5238.512	0.3	(K)/KPFHLVGT**S**MGGQVAGVYAAYYPSDVSSL CLVCPAGLQYSTDNQFVQR/(L)	130–177	2 CAM	0
3862.91	3862.964	13.9	(R)/LKELQGSAAVEKIPLIPSTPEEMSEMLQLCSY VR/(F)	178–211	CAM MSO	2
2593.31	2593.271	−15	(K)/IPLIPSTPEEMSEMLQLCSYVR/(F)	190–211	CAM	0
2609.27	2609.266	−1.5	(K)/IPLIPSTPEEMSEMLQLCSYVR/(F)	190–211	CAM MSO	0
1739.98	1740.017	21	(R)/FKVPQQILQGLVDVR/(I)	212–226		1
1464.89	1464.853	−25	(K)/VPQQILQGLVDVR/(I)	214–226		0
2247.2	2247.228	12.6	(R)/IPHNNFYRKLFLEIVSEK/(S)	227–244		2
1135.54	1135.52	−0.03	(R)/YSLHQNMDK/(I)	247–255		0
2628.38	2628.37	−3.7	(K)/VPTQIIWGKQ**D**QVLDVSGADMLAK/(S)	258–281	MSO	1
2698.18	2698.28	38.8	(K)/SIANCQVELLENCG**H**SVVMERPR/(K)	282–304	2 CAM	0
1813.94	1813.944	2.3	(K)/LIIDFLASVHNTDNNK/(K)	309–324		0

^*^Amino acids positions are based on recombinant hΔ29–3-ABHD6 protein sequence. A catalytic triad Ser^148^, Asp^278^ and His^306^ (positions of catalytic resides in WT hABHD6 sequence, is presented in bold).

**Figure 5:**
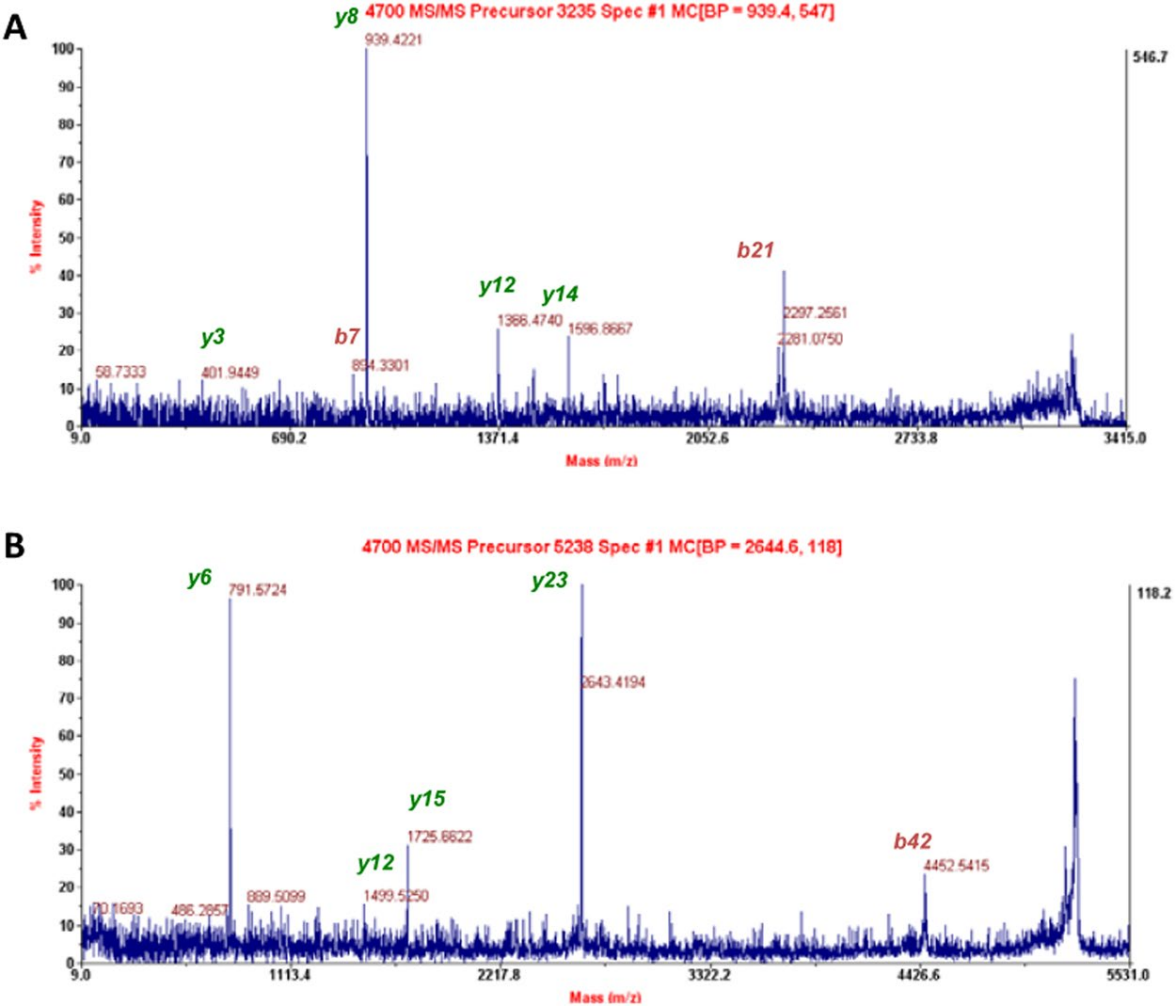
MS/MS fragmentation of selected peptide ions. (**A**) Matching expected fragments of the precursor ion with *m/z* 3235 for the sequence AHHHHHHVGTGSNDDDDKSPDPWPSALIR labeled in green and red. (**B**) Matching expected fragments of the precursor ion with *m/z* 5238 for the sequence KPFHLVGTSMGGQVAGVYAAYYPSDVSSLCVCPAGLQYSTDNQFVQR labeled in green and red.

We unambiguously identified a posttranslational modification of recombinant ABHD6 protein. Neither an *N*-terminal MAHHHHHHVGTGSNDDDDK (calculated *m*/*z* 2146.89) nor MAHHHHHHVGTGSNDDDDKSPDPWPSALIR (one missed cleavage; calculated *m*/*z* 3366.52) were observed in the trypsin digest of MALDI-TOF MS analyzed hΔ29-3-ABHD6 samples. Rather, an ion with *m*/*z* 3235.52 was invariably detected (Fig. 4) and after fragmentation (Fig. 5A) was identified as the peptide AHHHHHHVGTGSNDDDDKSPDPWPSALIR (one missed cleavage; calculated *m*/*z* 3235.48). It appears the *N*-terminal methionine was removed post-translationally from the expressed protein by methionyl aminopeptidase. A similar modification has been previously shown in recombinant hMGL^16^ and is likely to occur when the *N*-terminal methionine is followed by an alanine residue^24^. Interestingly an *N*-terminal, His6-tagged peptide AHHHHHHVGTGSNDDDDK (calculated *m*/*z* 2015.85) was not observed in the MALDI spectrum. Accordingly, Siepen *et al*. studies^25^ suggest that the negatively charged amino acid residues particularly at positions P4, P3, P1′ and P2′ relatively to lysine or arginine (P0) were in favor of trypsin missed cleavages. Therefore the observed missed cleavage was expected due to the presence at positions P4 and P3 of two consecutive aspartic acid residues preceding a basic lysine. The largest ion with *m*/*z* 5238.51 was observed (Fig. 4) and after fragmentation (Fig. 5B) was identified as the 48 amino acid peptide containing the catalytic serine (Ser^148^).

Together, the MALDI-TOF MS peptide fingerprint results accurately and confidently identified the purified recombinant protein as hΔ29-3-ABHD6 variant and provided confidence that the majority of individual tryptic peptides can be characterized during LAPS studies of this enzyme treated with selected inhibitors.

### MALDI-TOF MS Analysis of hABHD6 Inhibition by AM6701 and WWL70

**Figure 6:**
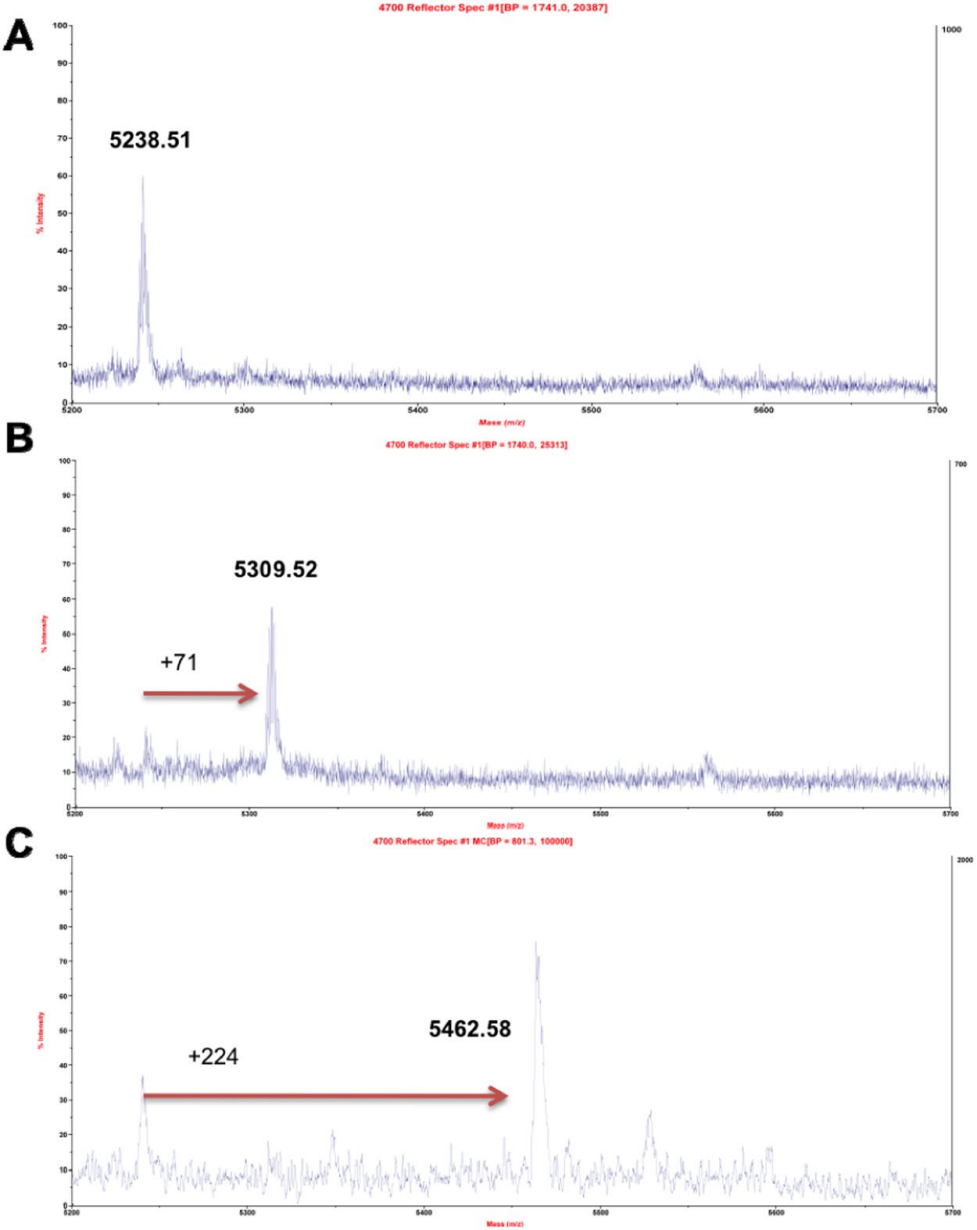
Ligand Assisted Protein Structure analysis with covalent inhibitors. Zoomed area *m/z* 5200–5700 of trypsin digested hΔ29-3-ABHD6. (**A**) Spectrum of the peptide containing the active-site serine residue. (**B**) Spectrum of the peptide containing the active-site serine residue modified by AM6701. (**C**) Spectrum of the peptide containing the active-site serine residue modified by WWL70.

The active site of recombinant hABHD6 was characterized by the LAPS approach using MALDI TOF mass spectrometry and covalent probes: the ABHD6-selective inhibitor WWL70, and the potent non-selective serine hydrolase inhibitor AM6701. The interactions of purified hΔ29-3-ABHD6 with AM6701 or WWL70 (apparent *IC*_50_, 0.9 nM and 285 nM, respectively, Supplementary Fig. S4A,B) were conducted at 1 to 10 molar ratio of enzyme to inhibitor. After incubation for 1 hr at room temperature enzyme inactivation was evaluated using the fluorometric assay. A completely inactivated enzyme was reduced by DTT, alkylated with IAM, and desalted on a Bio-Spin 6 column in ammonium bicarbonate buffer (pH 8.0) containing MS friendly octyl-β-glucoside surfactant (0.05%) to prevent protein aggregation and precipitation. Prior to MALDI-TOF MS analysis, desalted protein samples were subjected to overnight trypsin digestion at 37 °C. The carbamylation products, as result of enzyme inhibition, may form by an attack of activated Ser^148^ hydroxyl group on the AM6701 or WWL70 carbonyl moiety, the resultant carbamylated serine will show a 71 Da or 224 Da increase in the mass of the peptide (Supplemental Fig. 4C). The active-site serine peptides with two carbamidomethylated cysteines in trypsin digested untreated and AM6701 or WWL70 treated hABHD6 samples were expected to be seen as single charged ion at 5238.51, 5309.55 (addition of the 71 Da) or 5462.61 *m*/*z* (addition of the 224 Da), respectively (Supplemental Fig. S4C). A comparison of the MS spectra of the tryptic digest from untreated and AM6701 or WWL70 inactivated hABHD6 revealed that the inhibitor-treated samples contained only one peptide with expected mass increase to 5309.52 *m*/*z* (5.5 ppm error, addition of 71 Da) or to 5462.58 *m*/*z* (4.9 ppm error, addition of 224 Da) (Fig. 6A–C), which was attributed to the carbamylation of peptide containing the catalytic Ser^148^ residue. The carbamylation products were sensitive to the standard dithiothreitol treatment at 56 °C for 1 hr, typically employed to disrupt disulfide bonds prior to alkylation with IAM. To prevent removal of the carbamyl groups from inhibited hABHD6, a limited 15 min dithiothreitol treatment at room temperature was conducted. Under these mild conditions ~80% and ~65% of the peptides carbamylation observed in AM6701 and WWL70 inhibited hABHD6 were retained, respectively (Fig. 6B,C). These data demonstrate the essential role of catalytic Ser^148^ in the conserved GXSXG motif for hABHD6 esterase activity.

## Conclusion

We expressed, purified, and characterized a functional recombinant hABHD6 protein. The enzymatic parameters (*K*_m_ and *V*_max_) for hydrolysis of the native substrate, 2-AG, by recombinant hABHD6 in two different biochemical assays were shown to be comparable to values previously reported in the literature for the enzyme expressed in mammalian cells^6^. In addition we developed a high-throughput fluorescent assay based on the fluorogenic substrate AHMMCE for the reliable, fast and efficient screening of compound libraries aiming to identify hABHD6 inhibitors.

We found that truncation of the hABHD6 TM domain and additional elimination of three aromatic amino acid residues from aromatic patch Y^38^YWYW^42^ of the protein (hΔ29-3-ABHD6) increased soluble expression and significantly improved efficiency of IMAC purification without compromising the enzyme activity. This confirmed that the TM domain is not essential for hydrolysis and mainly serves to localize hABHD6 activity in close proximity to the membrane. The purified hΔ29-3-ABHD6 was digested with trypsin and peptides covering more than 95% of the hΔ29-3-ABHD6 sequence were observed in MALDI TOF MS spectra, including the largest 48 amino acid peptide and an *N*-terminal peptide without metionine indicating a post-translational modification in the recombinant protein. The mechanism of inhibition of hABHD6 by AM6701 or WWL70 was identified based on MS analysis of trypsin digested enzyme following LAPS approach as a selective enzyme carbamylation in the peptide containing the catalytic serine residue (Ser^148^). The production of active and pure recombinant hABHD6 is necessary for future structural analyses that will aid in the development of selective inhibitors of hABHD6 as drug candidates to treat disorders for which potentiating endocannabinoid-system activity might be therapeutic.

## Electronic supplementary material

Supporting information

## Data Availability

No datasets were generated or analyzed during the current study.
