# Supplementary material for: Biochemical and Proteomic Characterization of Recombinant Human α/β Hydrolase Domain 6
Source: Sci Rep. 2019 Jan 29;9:890. doi: 10.1038/s41598-018-36633-4 (PMC6351631; doi:10.1038/s41598-018-36633-4)
Supplement: Supplementary file 1 — Supporting information [file 41598_2018_36633_MOESM1_ESM.pdf]

SUPPORTING INFORMATION

Biochemical and Proteomic Characterization of Recombinant Human α/β Hydrolase Domain 6

Christina Miyabe Shields,[1,2] Nikolai Zvonok,[1,2]* Alexander Zvonok,[1,2] Alexandros Makriyannis[1,2,3]

[1] *Department of Pharmaceutical Sciences, Northeastern University, Boston, MA 02115, USA*

[2] *Center for Drug Discovery, Northeastern University, Boston, MA 02115, USA*

[3] *Department of Chemistry and Chemical Biology, Northeastern University, Boston, MA 02115, USA*

List of Supplementary components:

Supplemental Figure S1. SDS-PAGE analysis of full-length recombinant hABHD6 membrane fraction preparation and IMAC purification.

Supplemental Figure S2. Analysis activities of *N*- and *C*-terminal hexa-histidine tagged hΔ29-ABHD6 proteins in imidazole washes following purifications on Talon resin.

Supplemental Figure S3. SDS PAGE analysis of purification of C-terminal hexa-histidine hΔ29-ABHD6 protein with different detergents.

Supplemental Figure S4. Inhibition of recombinant hABHD6 by covalent inhibitors AM6701 **and WWL70.**

Unedited gels/blots.

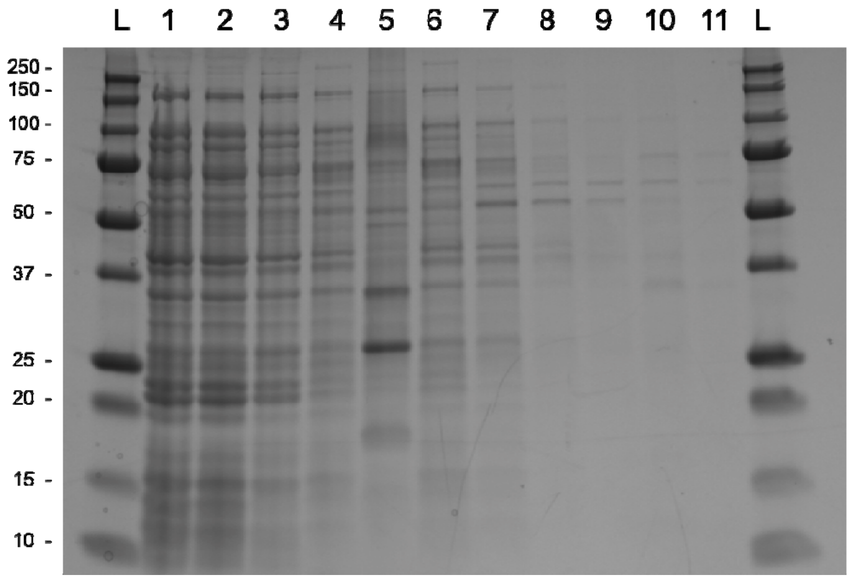

Supplemental Figure S1. SDS-PAGE analysis of full-length recombinant hABHD6 membrane fraction preparation and IMAC purification. Lanes are: (1) - total lysate, (2-4) - washes 1-3 of membranes, (5) - membrane fraction, (6) - unbound to Talon resin solubilized membrane fraction, (7-9) - Talon resin washes 1-3, (10) - elution from Talon resin, (11) - post-purified elution from Talon resin.

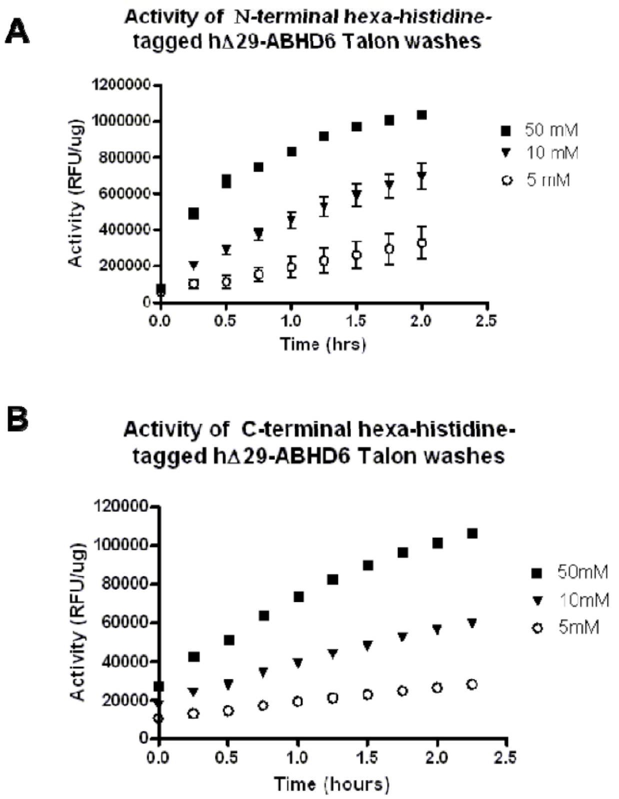

Supplemental Figure S2. Analysis activities of *N*- and *C*-terminal hexa-histidine tagged hΔ29-ABHD6 proteins in imidazole washes following purifications on Talon resin. Activity of *N*-terminal **(A)** and *C*-terminal (**B)** hexa-histidine tagged hΔ29-ABHD6 washes with imidazole concentrations of 5, 10 and 50 mM. All enzyme assays following purifications were performed in triplicate.

Methods

A cell pellet (2 g) with either full-length hABHD6 or hΔ29-3-ABHD6 was resuspended in 25 mL of Lys1 buffer (150 mM NaCl, 50 mM Tris, pH 8.0) containing 1% Triton X-100 and sonicated on ice using three, 50 s sonication cycles: 1s sonication bursts at 50 W power separated by 5 s intervals. The lysate was rotated at 4 ^{o}C for 1 hr to complete extraction and solubilization of proteins from cells debris and then centrifuged at 20,000 x g for 10 min at 4 ^{o}C. The

supernatant was diluted 1:1 with Lys1 buffer and incubated for 1 hr with 200 μL (bead volume) of pre-equilibrated BD Talon metal-affinity resin (Clontech, Mountain View CA) at room temperature. The suspension was transferred to a gravity-flow column and allowed to settle. The resin was washed once with 20 mL of Lys1 buffer containing 0.5% Triton X-100, and 5 mM imidazole, once with 20 mL of Lys1 buffer containing 0.5% Triton X-100 and 10 mM imidazole, and once with Lys1 buffer containing 0.5% Triton X-100 and 50 mM imidazole. The washes were saved and a total protein concentration was measured with the Pierce 660 Kit (Thermo Fisher Scientific, Pittsburgh, PA). A Synergy HT (BioTek Instruments, Winooski, VT) or EnVision (Perkin Elmer, Waltham, MA) plate readers were used in a 96-well plate format assays to measure protein concentrations or fluorescence of formed product.

Sample enzyme from washes (1 μg of total protein in each well of a 96-well Costar 3650 plate) in 50 mM Tris-HCl, pH 7.6 assay buffer (AB) was incubated with 20 μM of AHMMCE for 1 hr at room temperature while the fluorescence was read every 15 min following excitation at 360 nm and emission at 460 nm (λ_{ex}360 nm /λ_{em}460 nm). The relative fluorescence units were converted to the concentration of 7-hydroxy-6-methoxy-4-methylcoumarin (HMMC) formed based on a standard curve of HMMC. Data was normalized relatively to a negligible non enzymatic AHMMCE hydrolysis and used for calculation of the hABHD6 enzyme kinetic parameters.

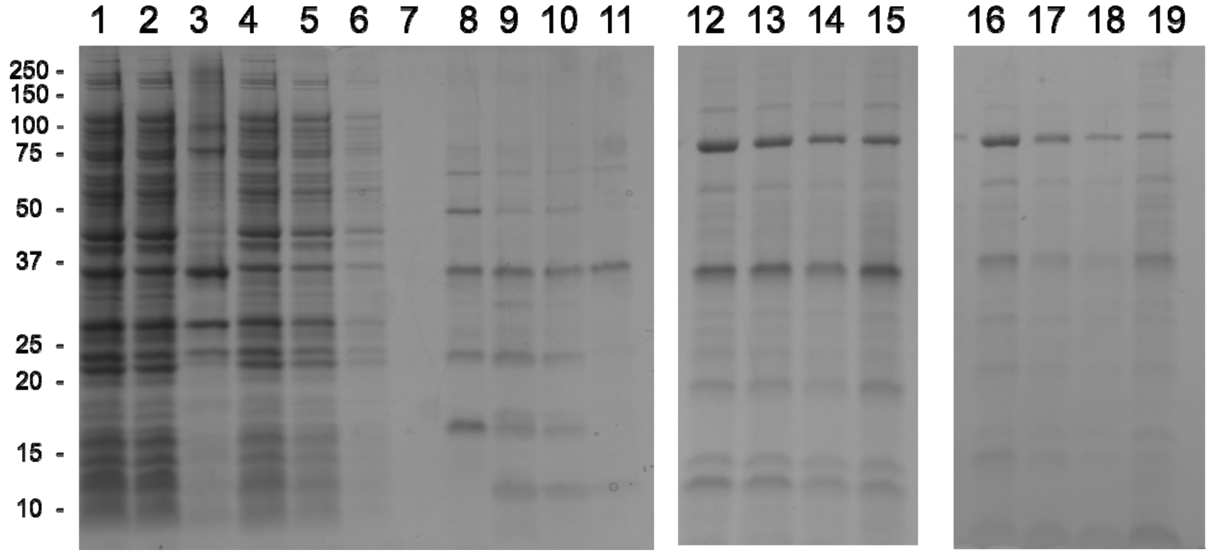

Supplemental Figure S3. SDS PAGE analysis of purification of C-terminal hexa-histidine hΔ29-ABHD6 protein with different detergents (Triton X-100 in lines 1-11, X-Tractor buffer in lines 12-15, and OBG in lines 16-19). Lanes are: (1) - total lysate, (2) - soluble fraction, (3) - insoluble fraction, (4) - unbound to resin fraction, (5-7) - resin washes 1-3, (8-10) - eluted fractions 1, 3 and 5; (12-14) - eluted fractions 1, 3 and 5, (16-18) - eluted fractions 1, 3 and 5; (11, 15 and 19) - Talon resin wash with 200 mM EDTA following imidazole elutions.

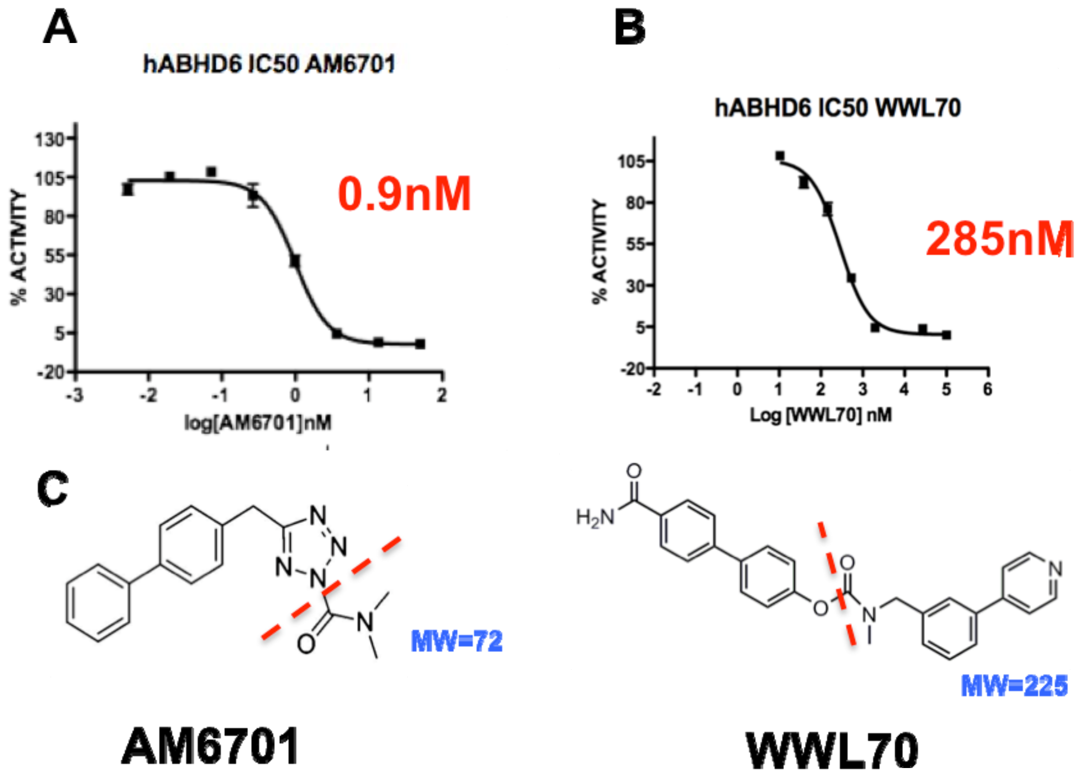

Supplemental Figure S4. Inhibition of recombinant hABHD6 by covalent inhibitors AM6701 and WWL70. Representative 8-point inhibition curves and apparent IC_{50} values for AM6701 **(A)** and WWL70 **(B)**. Structure of inhibitors and attachment groups with mass additions to the active-site serine residue proposed for MALDI-TOF MS data analysis **(C)**.

Unedited gels/blots:

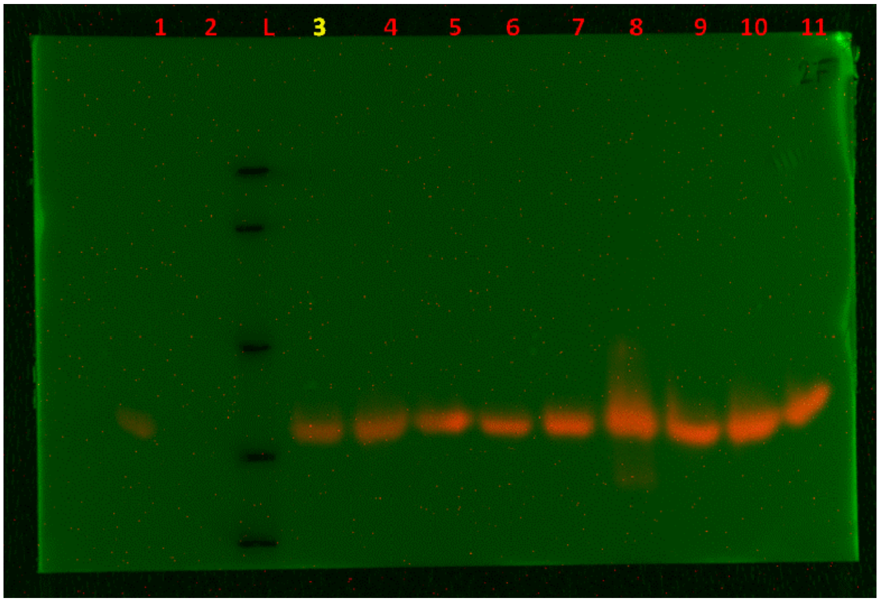

Figure 1. Full-length recombinant hABHD6 enzyme purification. A) Western Blot analysis of the recombinant hABHD6 purification using anti-mABHD6 antibody. Lanes are: (1) - total lysate, (2) - soluble fraction, (3-7) - Talon resin washes 1-5, (8) - insoluble fraction, (9-11) - fractions 1-3 of eluted hABHD6 from Talon resin.

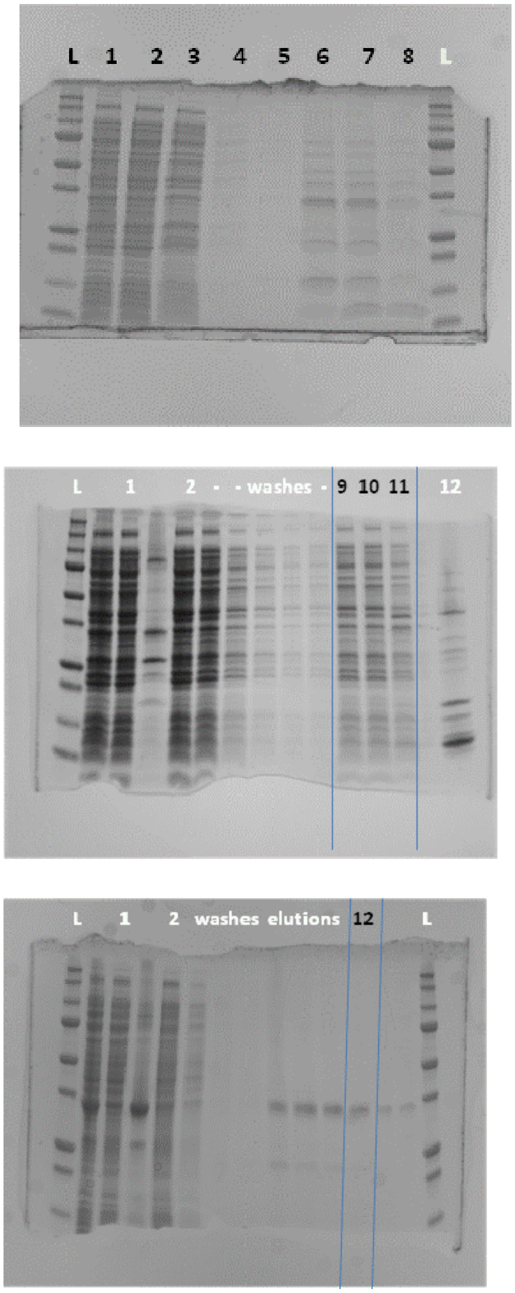

Figure 2. Recombinant Δ29, 37 and 42 hABHD6 truncated variants characterization. B) SDS-PAGE analysis of purification of hΔ29-ABHD6: using Talon Co^{2+} resin, (1) - soluble cells lysate, (2) - unbound to resin fraction, (3-5) – resin washes 1-3, (6-8) - imidazole elutions 1, 3 and 5; and using cOmplete Ni^{2+} resin, (9-11) - imidazole elutions 1, 3 and 5, (12) - Talon resin 200 mM EDTA wash following imidazole elutions.

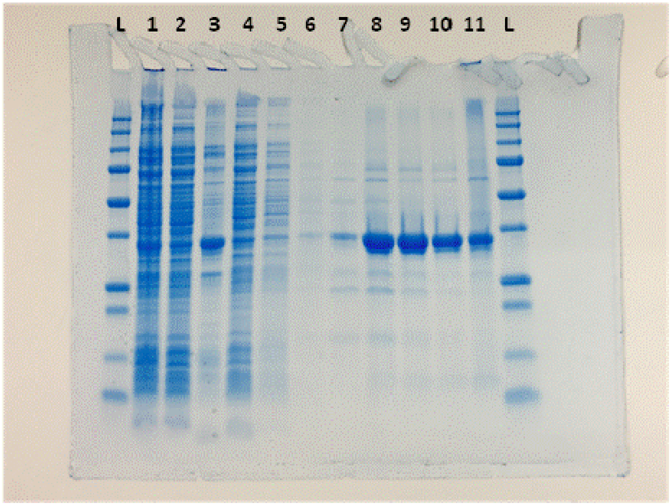

Figure 3. The hΔ29-3-ABHD6 variant characterization. D) Purification of hΔ29-3-ABHD6. Lanes are: (1) - total lysate, (2) - soluble fraction, (3) - insoluble fraction, (4) - unbound to resin fraction, (5-7) - resin washes 1-3 and (8-11) - elutions 1-4.

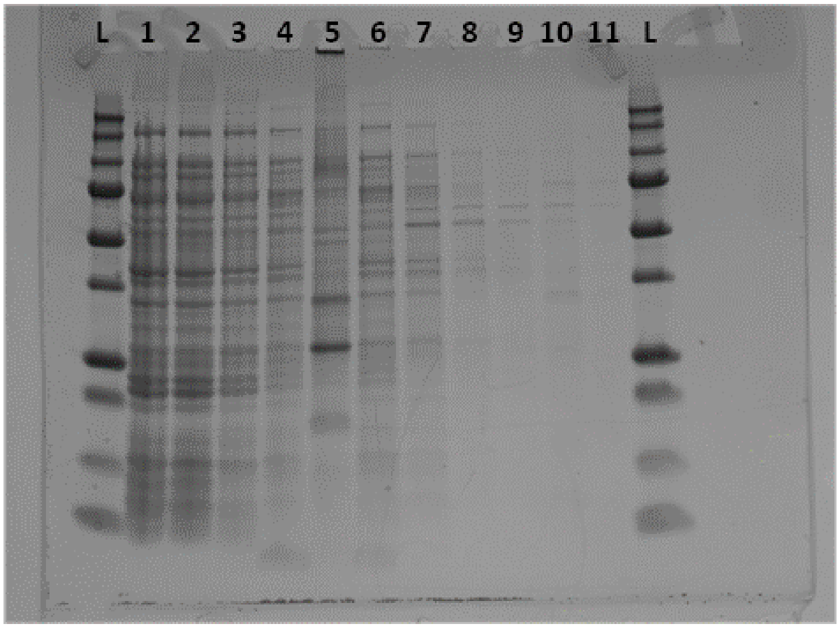

Supplemental Figure S1. SDS-PAGE analysis of full-length recombinant hABHD6 membrane fraction preparation and IMAC purification. Lanes are: (1) - total lysate, (2-4) - washes 1-3 of membranes, (5) - membrane fraction, (6) - unbound to Talon resin solubilized membrane fraction, (7-9) - Talon resin washes 1-3, (10) - elution from Talon resin, (11) - post-purified elution from Talon resin.

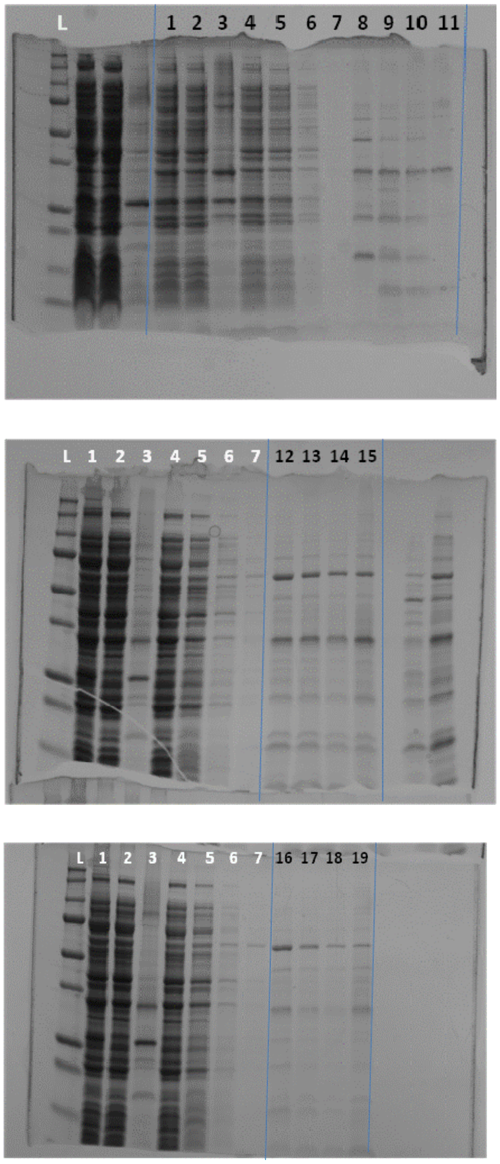

Supplemental Figure S3. SDS PAGE analysis of purification of C-terminal hexa-histidine hΔ29-ABHD6 protein with different detergents (Triton X-100 in lines 1-11, X-Tractor buffer in lines 12-15, and OBG in lines 16-19). Lanes are: (1) - total lysate, (2) - soluble fraction, (3) - insoluble fraction, (4) - unbound to resin fraction, (5-7) - resin washes 1-3, (8-10) - eluted fractions 1, 3 and 5; (12-14) - eluted fractions 1, 3 and 5, (16-18) - eluted fractions 1, 3 and 5; (11, 15 and 19) - Talon resin wash with 200 mM EDTA following imidazole elutions.
